# Evaluating the impact of neighborhood socioenvironmental burden on patient characteristics and survival in liver transplant recipients

**DOI:** 10.3389/frtra.2026.1731241

**Published:** 2026-04-09

**Authors:** Samar Semaan, Ashton A. Connor, Sudha Kodali, Anil K. Vadathya, Ahmed Elaileh, Youssef Dib, Khush Patel, Jason Todd, Jennifer Cullen, Caroline J. Simon, Yee Lee Cheah, Constance M. Mobley, Ashish Saharia, Tamneet Basra, David W. Victor, Linda W. Moore, A. Osama Gaber, R. Mark Ghobrial

**Affiliations:** 1 Department of Surgery, Houston Methodist Hospital, Houston, TX, United States; 2 JC Walter Jr Transplant Center, Houston Methodist Hospital, Houston, TX, United States; 3 Department of Surgery, Weill Cornell Medical College, New York, NY, United States; 4 Sherrie and Alan Conover Center for Liver Disease and Transplantation, Houston Methodist Hospital, Houston, TX, United States; 5 Department of Medicine, Weill Cornell Medical College, New York, NY, United States; 6 Houston Methodist Academic Institute, Houston, TX, United States; 7 Dr. Mary and Ron Neal Cancer Center, Houston Methodist Hospital, Houston, TX, United States

**Keywords:** environmental justice index, graft survival, liver transplant, overall survival, social determinants of health

## Abstract

**Background:**

The Environmental Justice Index (EJI) measures neighborhood level environmental, social, and health disparities. The Social-Environmental Ranking (SER) components of the EJI is used when linking to health outcomes. Higher SER scores (≥0.75) indicate greater environmental injustice and potential health inequities. This study assesses whether EJI's through SER can inform the care of liver transplantation (LT) patients in Houston, Texas.

**Methods:**

This single-center, retrospective analysis was conducted on LT recipients between January 2008 and December 2024. Patient addresses were linked to census tract-level EJI data and stratified at a 0.75 score threshold. Propensity score matching (PSM) was performed. Overall survival (OS) was assessed pre and post matching.

**Results:**

A total of 2,030 LT recipients were stratified by SER score (<0.75 vs. ≥0.75). Pre-matching, high SER patients were more often female (44.4% vs. 38.9%, *p* = 0.02), non-Hispanic Black (14.9% vs. 6.7%) or Hispanic (36.2% vs. 16.1%, *p* < 0.001), had public insurance (44.0% vs. 33.8%, *p* < 0.001), were unemployed at transplant (27.4% vs. 31.9%, *p* = 0.046), and had lower education levels (*p* < 0.001). They also had higher BMI (28.90 vs. 28.05, *p* = 0.024), longer waitlist times (103 vs. 71 days, *p* = 0.036), more diabetes (35.4% vs. 28.4%, *p* = 0.002), CMV positivity (79.3% vs. 71.0%, *p* < 0.001), and multi-organ transplants (18.6% vs. 14.4%, *p* = 0.015). Pre-LT, more were hospitalized on the floor (21.8% vs. 14.0%) and fewer in ICU (40.3% vs. 44.4%, *p* < 0.001). PSM yielded 619 matched pairs with covariate balance (SMD < 0.1). OS and graft survival did not differ by SER strata before or after matching (*p* > 0.05).

**Conclusion:**

These findings suggest that EJI is associated with pre-transplant variables and may help identify patients in need of social supports. However, individual patient factors seem to determine post-LT survival.

## Introduction

Liver transplantation (LT) remains the only curative treatment for patients with end-stage liver disease. LT outcomes and access, however, vary between populations depending on social determinants of health (SDOH) such as socioeconomic status, insurance, and education (1). Recently, neighborhood-level indices have become increasingly popular as a way to quantify community-level disadvantages and their impact on health outcomes (2). The Environmental Justice Index is one such score that measures neighborhood level environmental, social, and health disparities (3).

A growing body of literature is addressing the association between LT outcomes and poorer neighborhood level scores (4–6) However, while the EJI has been linked to outcomes' disparities in chronic diseases, cancer and other field of solid organ transplantation, it has not yet been studied in the context of liver transplantation (7–9) Moreover, in the Houston–South Texas region, concentrated industrial activity disproportionately affects African American and Hispanic neighborhoods, where these minority communities experience greater disparities in access to clean water, clean air, and healthcare services (10, 11).

This study evaluates the association of the EJI score with peri-LT patient variables and on post-procedure survival at a single center in Houston, Texas. The aim is to assess whether EJI can identify patients from high-burden communities who may benefit from targeted support initiatives, and if neighborhood-level burden independently predicts post-LT survival.

## Methods

### Study design and population

This study is a retrospective, single-center cohort study of adult LT recipients between January 2008 and December 2024 in the Houston, Texas area. Research was conducted under the center's Institutional Review Board Protocol Pro00000587, and requirement for informed was waived. Patients with at least one year of follow-up and verifiable survival status were included. Patients lacking address data were excluded. Demographic, clinical, and transplant-related variables were extracted from institutional databases. For patients transplanted more than once, demographic data was collected only from the index transplant, and overall survival (OS) was measured from the index transplant date to the date of last follow up or death.

### Environmental and social risk exposure

Patient addresses recorded at referral were used for exposure assignment. Patient addresses were geocoded and linked to census tract-level GEOIDs. The GEOIDs were then matched to 2022 data from the Center for Disease Control's (CDC) EJI scores. The three EJI modules are *Environmental Burden*, *Social Vulnerability*, and *Health Vulnerability,* and each of them contains specific sub-domains (3, 12). For the purpose of this study, the combined *Environmental Burden* and *Social Vulnerability* modules, the Social-Environmental Ranking (SER) score, were used, as the Center for Disease Control (CDC) recommends using the SER when linking to health outcomes (13). Accordingly, we utilized the SER sub-score to stratify our cohort, using the upper quartile cut-point to isolate the highest exposure segment, consistent with CDC interpretive convention and for analytic clarity. The SER score represents neighborhood-level environmental burden in addition to social disadvantage, reflecting factors like unemployment, poverty, low educational level, and housing instability. Higher EJI and SER scores (≥0.75) indicate greater environmental injustice, reduced access to healthcare and healthy living conditions, and exposure to hazardous environment, potentially leading to health inequities and increased social economic risk.

This threshold aligns with the EJI methodology, which often categorizes scores into quartiles for comparative analyses (14). To evaluate whether our results were dependent on this threshold, additional analyses were conducted using decile-based stratification, paired validation cohort comparisons, and treating SER as a continuous variable within Cox regression models. These approaches similarly showed no statistically significant association between SER and post-transplant outcomes, supporting that findings were not threshold dependent.

### Statistical analysis

Baseline characteristics were compared using parametric and non-parametric tests, as appropriate. Survival analysis was conducted to evaluate post-transplant outcomes and compare survival distributions between SER groups, with both log-rank test and weighted log-rank test highlighting long term outcomes used for statistical comparison. A two-sided *p*-value <0.05 was considered statistically significant. All statistical analyses were performed using R software. We used a complete case approach. Patients missing the exposure variable, missing survival time, or missing survival status were excluded from analysis. For multivariable models, individuals with missing values in included covariates were removed rather than imputed, and missingness was retained descriptively where relevant. Sensitivity analyses were performed to assess robustness of findings across alternative exposure categorizations of SER and with SER as a continuous variable.

### Propensity score matching

Propensity score matching was performed using a 1:1 nearest-neighbor algorithm without replacement. Matching covariates included race/ethnicity, education, payer at transplant, gender, and employment status. Covariate balance was assessed using standardized mean differences before and after matching. Matching and balance diagnostics were implemented using the MatchIt and cobalt packages in R. The matched dataset was then used for secondary analyses, including subgroup survival evaluation.

## Results

### Cohort features

**Figure 1:**
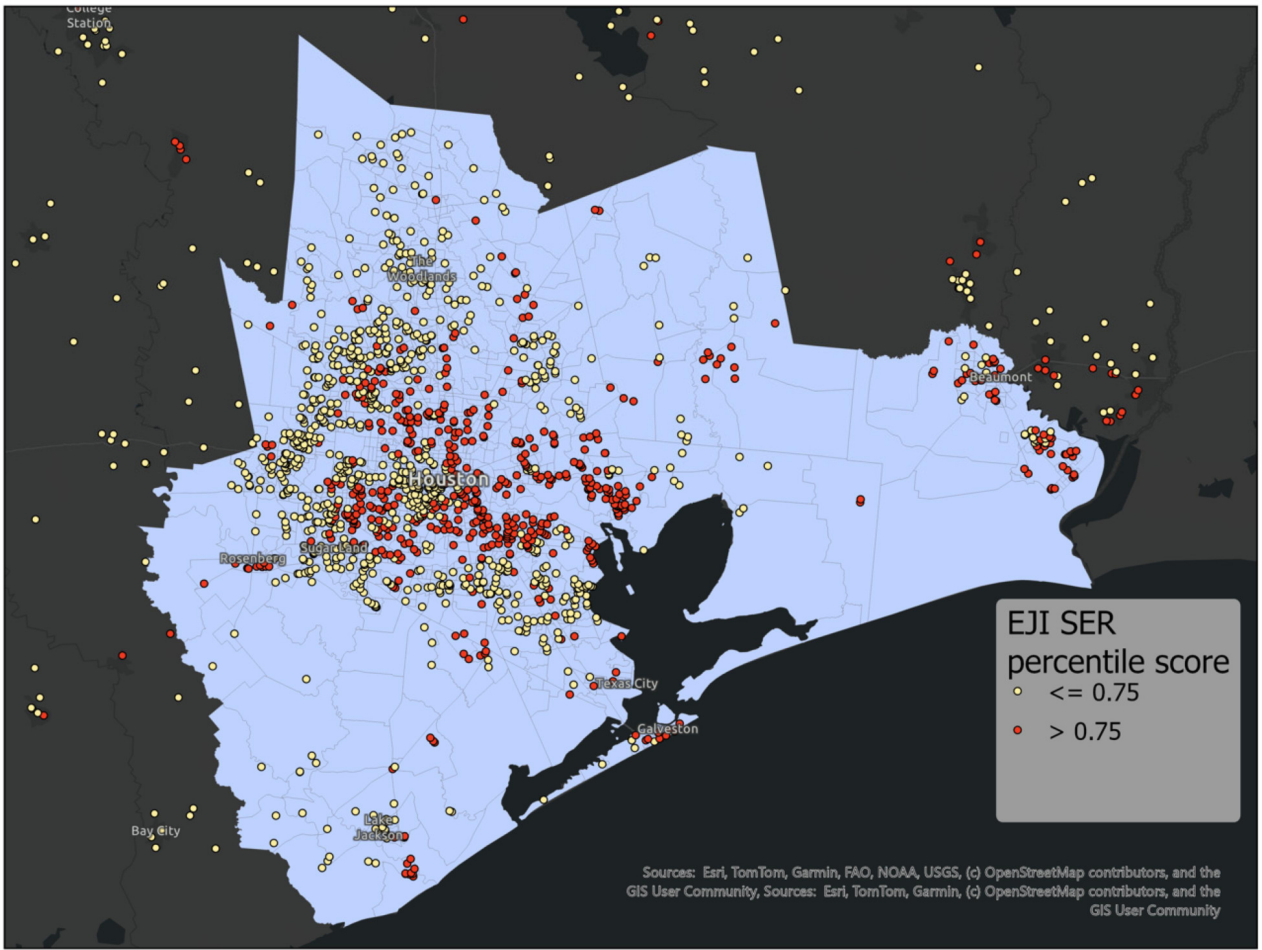
Geographic distribution of patients in the Houston, Texas catchment area stratified by SER score.

Our cohort includes 2030 patients that underwent LT between 01/2008 and 12/2024. There were 687 patients with an SER score of 0.75 or above, categorized as SER-H (33.8%), and 1343 had a score below 0.75, categorized as SER-L (66.2%). Demographic features (Table 1) include median age 58 years old (IQR: 49–65), median BMI: 28.33 (IQR: 24.61–32.95) kg/m^2^, and median biologic MELD at transplant of 29 (IQR: 16–37). Patients were mostly male (59.2%) and non-Hispanic White (63.4%). Median follow-up post transplantation was 1,218.5 days (IQR: 481.8–2419.8). Figure 1 shows the geographic distribution of our patients in the regional catchment area.

**Table 1:** Demographics table stratified by SER score—unmatched cohort.

Characteristics		Overall (*N* = 2,030)	SER ≥0.75 (*N* = 687)	SER <0.75 (*N* = 1,343)	*p*-Value
Gender (%)	Female	828 (40.8)	305 (44.4)	523 (38.9)	0.02
Race/ethnicity (%)	Non-Hispanic White	618 (49.9)	318 (46.4)	965 (72.1)	<0.001
	Non-Hispanic Asian	24 (1.9)	14 (2.0)	63 (4.7)	
	Non-Hispanic Black	173 (14.0)	102 (14.9)	90 (6.7)	
	Hispanic	416 (33.6)	248 (36.2)	216 (16.1)	
	Native American	7 (0.6)	3 (0.4)	4 (0.3)	
Age (median [IQR])		58.00 [49.00, 65.00]	59.00 [51.00, 65.00]	58.00 [47.50, 65.00]	0.083
BMI (median [IQR])		28.33 [24.61, 32.95]	28.90 [24.94, 33.46]	28.05 [24.37, 32.74]	0.024
MELD (median [IQR])		29.00 [16.00, 37.00]	29.00 [16.50, 36.00]	28.00 [16.00, 37.00]	0.886
CMV status (%)	Positive	1,479 (73.8)	541 (79.3)	938 (71.0)	<0.001
Diabetes diagnosis (%)	D.M. Type I	20 (1.0)	6 (0.9)	14 (1.1)	0.002
	D.M. Type II	547 (27.2)	209 (30.7)	338 (25.4)	
	D.M. Type Unknown	51 (2.5)	26 (3.8)	25 (1.9)	
	None	1,392 (69.3)	440 (64.6)	952 (71.6)	
Highest level of education (%)	Secondary	691 (36.5)	292 (46.3)	399 (31.6)	<0.001
	Post-Graduate	141 (7.4)	22 (3.5)	119 (9.4)	
	Primary	90 (4.8)	52 (8.2)	38 (3.0)	
	Trade	550 (29.0)	168 (26.6)	382 (30.2)	
	Undergraduate	422 (22.3)	97 (15.4)	325 (25.7)	
Condition at transplant (%)	Home	807 (40.3)	258 (37.8)	549 (41.6)	<0.001
	Hospital	334 (16.7)	149 (21.8)	185 (14.0)	
	ICU	861 (43.0)	275 (40.3)	586 (44.4)	
Insurance type (%)	Private	1,250 (62.7)	380 (56.0)	870 (66.2)	<0.001
	Public	744 (37.3)	299 (44.0)	445 (33.8)	
Employment status (%)	Employed	597 (30.4)	185 (27.4)	412 (31.9)	0.046
Cancer treatment (%)	LRT—TACE	177 (8.7)	67 (9.8)	110 (8.2)	0.273
	LRT—TARE	109 (5.4)	38 (5.5)	71 (5.3)	0.899
	LRT—Ablation	117 (5.8)	38 (5.5)	79 (5.9)	0.825
	LRT—Radiation	38 (1.9)	4 (0.6)	34 (2.5)	0.004
	Neoadjuvant systemic	128 (6.3)	34 (4.9)	94 (7.0)	0.089
	Adjuvant systemic	78 (3.8)	23 (3.3)	55 (4.1)	0.48
	Palliative systemic	38 (1.9)	11 (1.6)	27 (2.0)	0.638
Cancer diagnosis (%)	None	1,389 (68.4)	460 (67.0)	929 (69.2)	0.054
	CCA	71 (3.5)	16 (2.3)	55 (4.1)	
	Colorectal	5 (0.2)	1 (0.1)	4 (0.3)	
	HCC	537 (26.5)	200 (29.1)	337 (25.1)	
	Mixed HCC/CCA	22 (1.1)	6 (0.9)	16 (1.2)	
	Other	6 (0.3)	4 (0.6)	2 (0.1)	
Multi organ transplant (%)	Yes	321 (15.8)	128 (18.6)	193 (14.4)	0.015
Waitlist time in days (median [IQR])		84.00 [9.00, 354.75]	103.00 [11.00, 375.00]	71.00 [8.00, 341.50]	0.036
Graft survival in days (median [IQR])		1,051.00 [359.00, 2,186.25]	1,022.00 [360.00, 2,168.75]	1,061.50 [358.25, 2,189.00]	0.664

### Demographic differences by EJI score

There were significant socioeconomic differences in the pre-transplant variables between the SER-H and SER-L cohorts (Table 1). Patients in the SER-H group had higher BMI at transplantation (-H: 28.90, -L: 28.05, *p* = 0.024) and included more female patients (-H: 44.4%, -L: 38.9%, *p* = 0.02). Racial distribution varied considerably among groups, with SER-H group having more Hispanic (-H: 36.2%, -L: 16.1%) and non-Hispanic Black (-H: 14.9%, -L: 6.7%), and less Asian (-H: 2.0%, -L: 4.7%) and non-Hispanic White (-H: 46.4%, -L: 72.1%) representation (*p* < 0.001). More patients in the SER-L group possessed undergraduate (-H: 15.4%, -L: 25.7%) and post-graduate (-H: 3.5%, -L: 9.4%) level degrees, while those in the SER-H group had more secondary education (-H: 46.3%, -L: 31.6%) (*p* < 0.001). More individuals from the SER-H cohort were unemployed at the time of transplantation (-H: 27.4%, -L: 31.9%, *p* = 0.046). Insurance varied among groups, with SER-H patients possessing more public insurance (Medicare, Medicaid) (-H: 44.0%, -L: 33.8%), and SER-L patients being more often covered by private insurance (-H: 56.0% -L: 66.2%, *p* < 0.001).

### Key clinical differences

Clinically, several factors differed between cohorts (Table 1). SER-H patients were more likely to be positive for CMV antibodies (-H: 79.3%, -L: 71.0%, *p* < 0.001) and to have diabetes (-H: 35.4%, -L: 28.4%, *p* = 0.002). At transplantation, patients in the SER-H group were more likely to be hospitalized on the floor (-H: 21.8%, -L: 14.0%), but more patients in the SER-L group were admitted in the ICU (-H: 40.3%, -L: 44.4%, *p* < 0.001). Liver cancer is one of the indications for transplantation and can have two histological types: Hepatocellular carcinoma (HCC) and cholangiocarcinoma (CCA). HCC trended towards being more common in SER-H patients (-H: 29.1%, -L: 25.1%, *p* = 0.054), while CCA was slightly increased in the SER-L populations, but this was not significant (-H: 2.3%, -L: 4.1%, *p* = 0.054). SER-H patients with liver cancers also received less often pre-LT external beam radiation therapy (-H: 0.6%, -L: 2.5%, *p* = 0.004), while usage of other ablative and loco-regional therapies and systemic therapies pre-LT did not differ. More individuals in the SER-H group received multiorgan transplants (-H: 18.6%, -L: 14.4%, *p* = 0.015). The SER-L had significantly shorter median waitlist times (-H: 103 days, -L: 71 days, *p* = 0.036). There were no significant differences in MELD score among groups.

### Post matching results

Propensity score matching (PSM) at the 0.75 SER cutoff yielded 619 matched pairs, with a final cohort of 1,238 patients (Table 2). Median age was 59 years old (IQR: 50–65), median BMI 28.63 (24.91–33.48) kg/m^2^, and median biologic MELD at transplant of 28 (IQR: 16–36). Patients were still mostly male (55.5%) and non-Hispanic white (49.9%), with Hispanics being the second largest racial/ethnic group (33.6%). Median follow-up post transplantation was 1,158.5 (IQR: 498.25–2,341). There were no significant differences between cohorts in terms of gender, employment, insurance type and education. Race/ethnicity was still significantly different among groups, but differences were less pronounced (*p* = 0.038, Table 2). There were no other significant differences between SER-H and SER-L groups.

**Table 2:** Demographics table stratified by SER score—matched cohort.

Characteristics		Overall (*N* = 1,238)	SER ≥0.75 (*N* = 619)	SER <0.75 (*N* = 619)	*p*-Value
Gender (%)	Female	551 (44.5)	279 (45.1)	272 (43.9)	0.732
Race/ethnicity (%)	Non-Hispanic White	618 (49.9)	282 (45.6)	336 (54.3)	**0** **.** **038**
	Non-Hispanic Asian	24 (1.9)	13 (2.1)	11 (1.8)	
	Non-Hispanic Black	173 (14.0)	91 (14.7)	82 (13.2)	
	Hispanic	416 (33.6)	230 (37.2)	186 (30.0)	
	Native American	7 (0.6)	3 (0.5)	4 (0.6)	
Age (median [IQR])		59.00 [50.00, 65.00]	58.00 [50.00, 65.00]	59.00 [48.00, 65.00]	0.637
BMI (median [IQR])		28.63 [24.91, 33.48]	28.98 [25.06, 33.53]	28.21 [24.69, 33.14]	0.177
MELD (median [IQR])		28.00 [15.00, 36.00]	29.00 [15.00, 36.00]	28.00 [15.50, 36.00]	0.977
CMV status (%)	Positive	953 (77.0)	492 (79.5)	461 (74.5)	0.043
Diabetes diagnosis (%)	D.M. Type I	12 (1.0)	4 (0.6)	8 (1.3)	0.103
	D.M. Type II	368 (29.7)	196 (31.7)	172 (27.8)	
	D.M. Type Unknown	37 (3.0)	23 (3.7)	14 (2.3)	
	None	820 (66.3)	395 (63.9)	425 (68.7)	
Highest level of education (%)	Secondary	565 (45.6)	287 (46.4)	278 (44.9)	0.196
	Post-Graduate	49 (4.0)	22 (3.6)	27 (4.4)	
	Primary	87 (7.0)	52 (8.4)	35 (5.7)	
	Trade	324 (26.2)	162 (26.2)	162 (26.2)	
	Undergraduate	213 (17.2)	96 (15.5)	117 (18.9)	
Condition at transplant (%)	Home	501 (40.5)	239 (38.6)	262 (42.3)	0.035
	Hospital	229 (18.5)	132 (21.3)	97 (15.7)	
	ICU	508 (41.0)	248 (40.1)	260 (42.0)	
Insurance type (%)	Private	705 (56.9)	352 (56.9)	353 (57.0)	1
	Public	533 (43.1)	267 (43.1)	266 (43.0)	
Employment status (%)	Employed	355 (28.7)	179 (28.9)	176 (28.4)	0.9
Cancer treatment (%)	LRT—TACE	116 (9.4)	64 (10.3)	52 (8.4)	0.283
	LRT—TARE	67 (5.4)	36 (5.8)	31 (5.0)	0.615
	LRT—Ablation	74 (6.0)	37 (6.0)	37 (6.0)	1
	LRT—Radiation	14 (1.1)	3 (0.5)	11 (1.8)	0.06
	Neoadjuvant systemic	68 (5.5)	30 (4.8)	38 (6.1)	0.383
	Adjuvant systemic	45 (3.6)	21 (3.4)	24 (3.9)	0.761
	Palliative systemic	21 (1.7)	11 (1.8)	10 (1.6)	1
Cancer diagnosis (%)	None	833 (67.3)	414 (66.9)	419 (67.7)	0.361
	CCA	33 (2.7)	14 (2.3)	19 (3.1)	
	HCC	357 (28.8)	183 (29.6)	174 (28.1)	
	Mixed HCC/CCA	12 (1.0)	5 (0.8)	7 (1.1)	
	Other	3 (0.2)	3 (0.5)	0 (0.0)	
Multi organ transplant (%)	Yes	206 (16.6)	115 (18.6)	91 (14.7)	0.079
Waitlist time in days (median [IQR])		114.00 [11.00, 388.50]	125.00 [12.00, 390.00]	96.00 [10.50, 384.00]	0.515
Graft survival in days (median [IQR])		825.00 [360.25, 2,144.75]	859.00 [360.00, 2,113.50]	815.00 [362.00, 2,168.00]	0.743

Bold value is considered statistically significant (*p* < 0.05).

### Survival analysis

**Figure 2:**
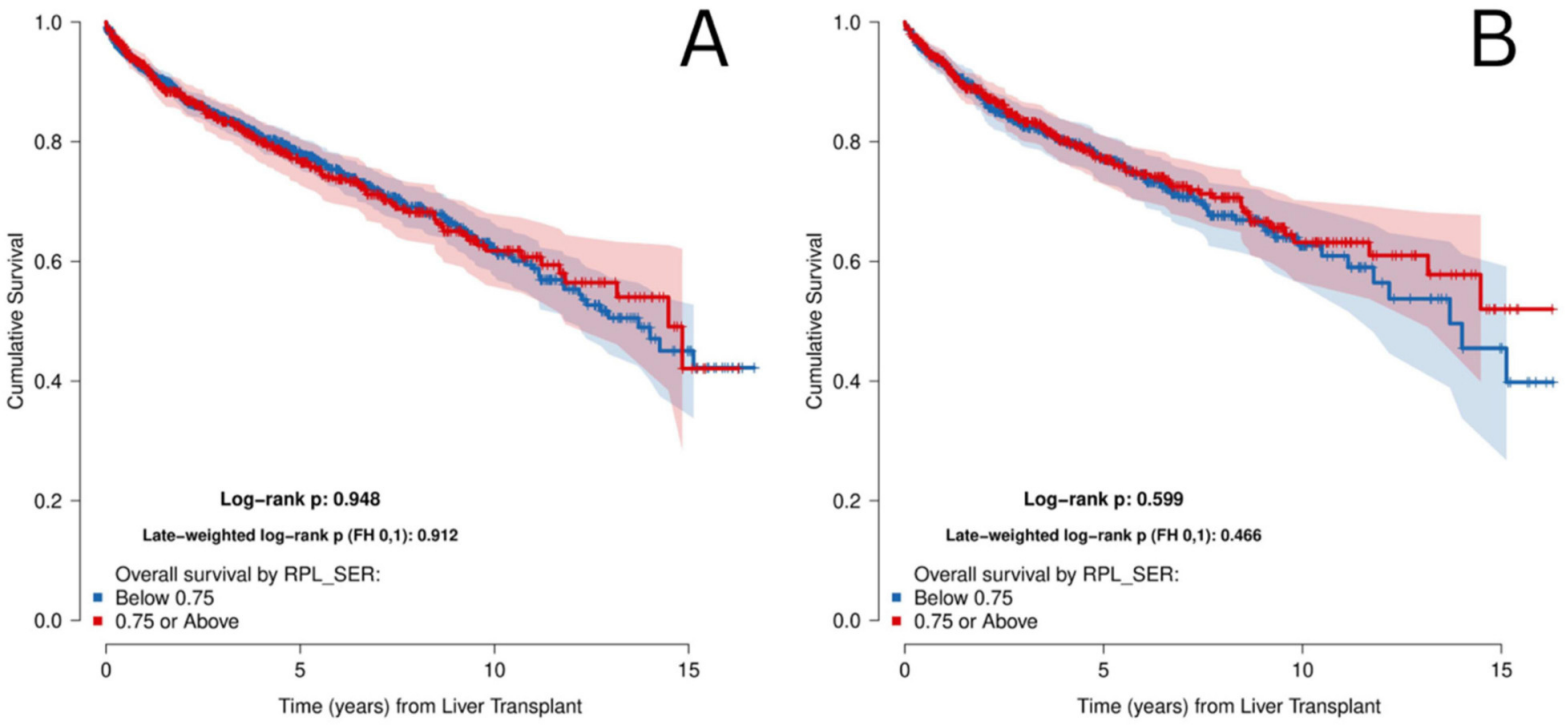
Overall survival from date of transplantation stratified by SER score in the **(A)** unmatched cohort and the **(B)** matched cohort.

**Figure 3:**
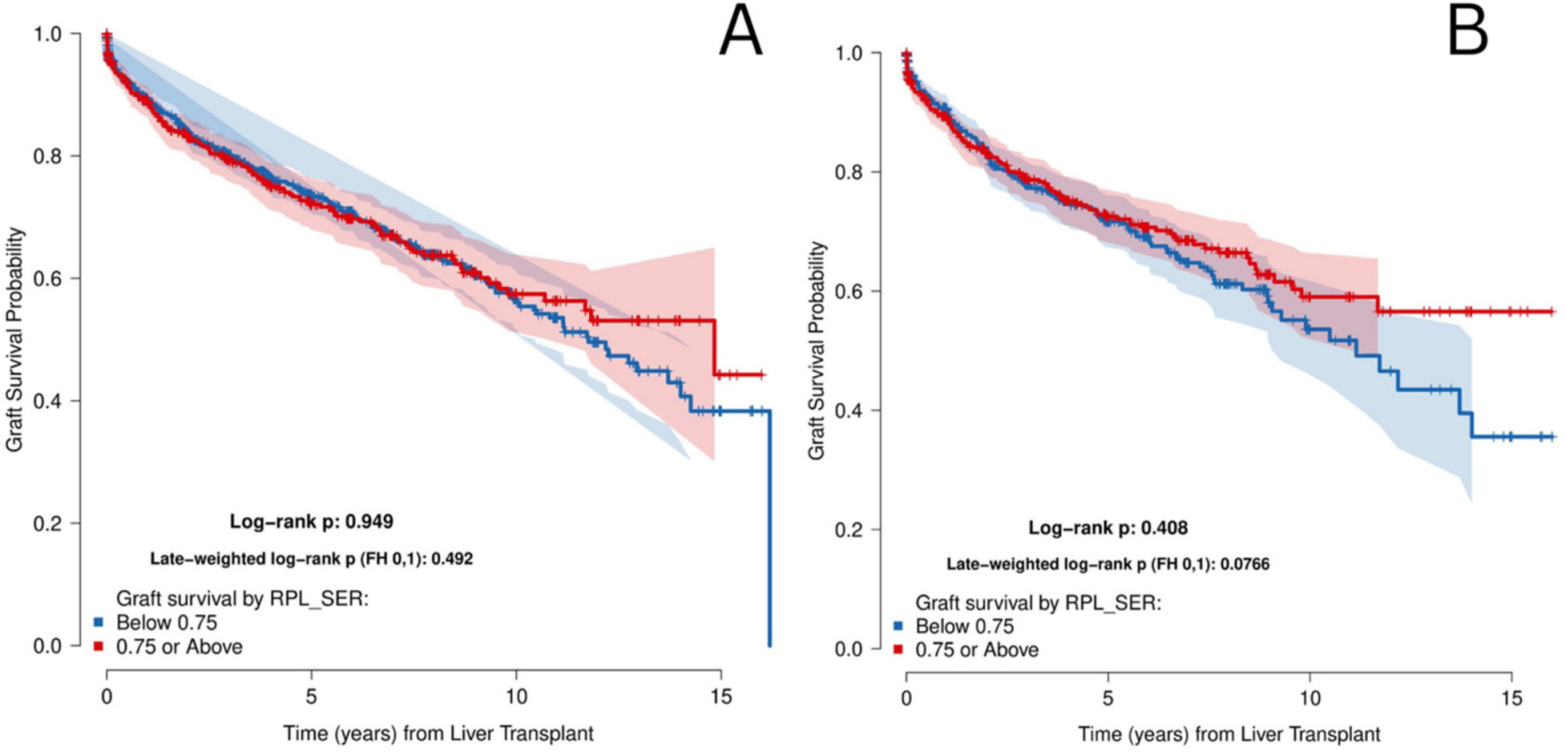
Index graft survival from date of transplantation stratified by SER score in the **(A)** unmatched cohort and the **(B)** matched cohort

In the unmatched cohort, median follow-up post-transplant was 1218.5 days (IQR: 481.8–2,419.8). Sensitivity analyses using multiple SER decile cutoff points were performed for both overall and graft survival and did not identify any threshold at which a statistically significant difference was observed (Supplementary Figures S1, S2). Probabilities of overall survival (OS) 1, 5, and 10-years post-LT were 92.3%, 77.5%, and 62% respectively (Supplementary Table S1). Probability of 5-year OS in the SER-H group was 76.5% compared to 78% in the SER-L group (Figure 2A). After matching, median follow-up was 1158.5 (IQR: 498.25–2,341), and probabilities of OS at 1, 5, and 10-years post-LT were 93.2%, 77.1%, and 62.9% respectively. 5-year OS probability in the SER-H group was 76.9%, compared to 77.1% in the SER-L group (Figure 2B). There was no statistically significant difference in OS between the two groups before or after matching, with both log-rank *p* and weighted log-rank *p* being >0.05 (Figure 2). Graft survival was also not significantly different between groups in both cohorts (*p* > 0.05) (Figure 3).

## Discussion

A growing body of literature has assessed how neighborhood-level indices are associated with outcomes of solid-organ transplant (SOT) patients. The Social Vulnerability Index (SVI), a census-track level score of social disadvantage, and Area Deprivation Index (ADI), a 1–100 composite socioeconomic score, are more frequently used than EJI (15, 16). The Environmental Justice Index (EJI) is a national, census-tract level measure of cumulative environmental, social, and health burden, developed by the CDC and ATSDR as the first place-based tool designed to quantify environmental injustice through a health equity lens (3). Unlike ADI or SVI, which primarily capture socioeconomic vulnerability, the EJI integrates environmental burden, social vulnerability, and health vulnerability, providing a more comprehensive assessment of neighborhood exposure risk (17).

In liver transplantation, higher SVI and ADI scores have been linked to lower likelihoods of waitlisting and transplantation, greater cirrhosis morbidity, and higher mortality, indicating that neighborhood disadvantage may influence outcomes throughout the transplant continuum (4, 5). Similar patterns have been reported in heart transplantation, where high SVI burden has been associated with increased rejection, rehospitalization, and reduced long-term survival, and in pediatric kidney transplant recipients, where children from highly deprived neighborhoods are less likely to receive preemptive or living-donor grafts (9, 18). National OPTN reports further highlight that ADI correlates with transplant activity across multiple organ systems (16). Collectively, these studies suggest that neighborhood-level disadvantage is associated with access to transplant and, in some cases, post-transplant outcomes. However, despite this emerging evidence base, no prior transplant studies have yet applied the Environmental Justice Index (EJI). While all neighborhood level indices share common features, the choice of index can impact outcomes and conclusions in transplantation cohorts, as highlighted by Park et al. (19). To our knowledge, based on a comprehensive review of the literature, this is the first study to apply the EJI to liver transplant outcomes.

### Interpretation of current study results

Our cohort was stratified into SER-H (SER ≥ 0.75) and SER-L (SER < 0.75) quartile-based categorization recommended for exploratory comparison of burdened vs. less burdened communities. We acknowledge that this threshold is not biologically meaningful and serves as a statistical tool rather than a definitive exposure cut-point (13).

Pre-matching, the SER-H group differed significantly in terms of socioeconomic and clinical characteristics, reflecting the characteristics of a vulnerable population. These differences mirror known patterns observed in the literature, where communities with the highest EJI scores typically comprise of more racial/ethnic minorities, lower income, and poorer housing and infrastructure (10, 20). Our SER-H population had higher percentages of Hispanic and Non-Hispanic Black individuals (*p* < 0.001), public insurance (*p* < 0.001), lower educational attainment (*p* < 0.001) and unemployment (*p* = 0.046). These pre-match imbalances highlight the ability of EJI scores to effectively flag patients living in structurally marginalized communities (4, 5).

Patients from socioeconomically disadvantaged neighborhoods face barriers throughout the liver transplant process. Studies have associated higher SVI scores with reduced likelihood of waitlisting and transplantation (4). Higher ADI scores have also been linked to more cirrhosis complications, increased mortality, and lower waitlisting rates (5). This is also apparent in different organ transplantation programs. Higher SVI and ADI score correlate with decreased access to transplantation and adverse transplantation outcomes in fields like heart and kidney transplantation (9, 18). The national OPTN SDOH report in 2024 linked ADI to transplant activity across organ types, highlighting the complexities and underscoring importance of the relationship between Neighborhood level scores and transplantation outcomes (16).

In recent years, however, EJI has also frequently been used as a measure of neighborhood level socio-environmental burden in association with various health outcomes. Recent studies have linked higher EJI scores with everything from decreased cancer screening, to worse colorectal cancer survival outcomes, as well as increased incidence of childhood asthma exacerbations and declining lung function (8, 20, 21).

To further evaluate whether the choice of quartile thresholds influenced our findings, we conducted sensitivity analyses, including decile-based evaluations, paired validation cohort comparisons, and modeling SER as a continuous variable via Cox regression. Across all approaches, we did not observe a statistically significant association between SER and overall or graft survival. This supports that the observed lack of association reflects the underlying relationship rather than dependence on a specific categorization strategy.

Our research, along with the available data, carry an important implication: EJI scores can be used pragmatically to identify vulnerable communities rather than as deterministic survival predictors. Within transplant centers, this may highlight at-risk patients requiring more intensive pre- and post-transplant support. Seeing as SER-H patients disproportionately display social vulnerabilities, programs could direct targeted outreach initiatives to support these individuals such as social work intervention, transportation or housing assistance, and health education. One example is a recent study by Kodali et al., which demonstrated that a culturally tailored Hispanic outreach program significantly increased liver transplant referrals and waitlisting at a single center, underscoring the potential impact of community-based engagement strategies on access to care (22). This could mitigate the non-medical risks that cluster in disadvantaged neighborhoods, as expressed in the litterature (18).

### Survival outcomes

After propensity score matching, the difference between SER-L and SER-H groups was drastically reduced. In both cohorts, survival curves for post-LT survival as well as long term graft survival were not significantly different between groups, with a log rank *p* > 0.05. This suggests that neighborhood EJI, or SER, does not appear to be an independent driver of post-transplant survival, and that patient-level characteristics and condition seem to play a stronger role.

This aligns with previously published literature highlighting the role of individual level clinical and socioeconomic factors on patient outcomes in different clinical fields (23, 24). A 2024 analysis on LT recipients from the united Network for Organ Sharing using Social Deprivation Index (SDI) score reported poorer post LT outcomes in association with individual socioeconomic factors, but no such significance with the neighborhood level SDI (1). Research also shows attenuated associations of ADI or social determinants with graft survival after accounting for immunologic risk and medical adherence in kidney transplantation.

Once patients with increased pre-transplant socioenvironmental barriers, as determined by EJI, become transplant recipients, standardized post-LT care pathways may help mitigate disparities, leading to comparable survival despite increased socio-environmental burden.

### Relevance to the Houston/South Texas context

Our transplant center serves a diverse population in Houston–South Texas, a region with significant environmental burden because of the high industrial activity. The neighborhood level impact and distribution of industrial burden disproportionately affects minority populations such as Hispanics and non-Hispanic Black people, who tend to live in higher EJI/SER score neighborhoods (10, 11). Historically burdened communities such as East/Southeast Houston and rural South Texas experience more water concerns, poorer air quality, and limited healthcare access. Research has even implied that while EJI scores may not be independent outcome drivers, they can lead to differential outcomes between minority and non-Hispanic White populations (11). Incorporating the EJI could help quantify neighborhood-level risk factors, offer more holistic understanding of geographic disparities, and contribute to the creation of community-focused outreach initiatives and interventions, as well as policy advocacy aimed at upstream determinants of health and transplant access.

### Limitations

Our study has several limitations. First, it is a single-center, retrospective cohort analysis, which may limit generalizability due to center-specific factors such as patient demographics (race/ethnicity) and pre- and post-transplantation protocols. The Environmental Justice Index (EJI) was assigned based on the earliest available patient addresses, which is our best estimate of pre-transplant neighborhood context. However, this assumes static residence and does not account for mobility or time spent in other areas. While propensity score matching improved group balance, unmeasured clinical differences may persist, and matching cannot fully adjust for unmeasured social or environmental confounders. Therefore, our findings should be interpreted as observational and hypothesis-generating.

We acknowledge limitations of categorizing EJI/SER; however, sensitivity analyses treating SER as a continuous variable yielded similar null findings, supporting the robustness of our results while recognizing that more granular modeling may still reveal nuanced effects. Future prospective or multi-center studies with more detailed environmental and social data are needed to validate these results. Additionally, incorporating more granular post-transplant information like adherence to follow-up, medication use, social support, and neighborhood dynamics, could better illuminate how EJI influences individual outcomes.

## Conclusion

This study is the first to apply the Environmental Justice Index (EJI) in liver transplantation. We found that patients with higher SER scores (≥0.75) had greater social and clinical vulnerability, underscoring the potential of EJI to identify at-risk populations. However, overall and graft survival after transplant did not differ significantly between high and low SER groups, suggesting that individual clinical factors, rather than neighborhood-level burden, play a larger and more significant role in post-transplant outcomes. EJI may still be a valuable tool for targeting pre-transplant support and guiding equity-focused interventions.

## Data Availability

The raw data supporting the conclusions of this article will be made available by the authors, without undue reservation.

## References

[1] Gummaraj Srinivas N Chen Y Rodday AM Ko D. Disparities in liver transplant outcomes: race/ethnicity and individual- and neighborhood-level socioeconomic status. Clin Nurs Res. (2024) 33(7):509–18. 10.1177/10547738241273128 39192612 PMC11421193

[2] Kind AJH Buckingham WR. Making neighborhood-disadvantage metrics accessible—the neighborhood atlas. N Engl J Med. (2018) 378(26):2456–8. 10.1056/NEJMp1802313 29949490 PMC6051533

[3] U.S. Department of Health and Human Services. Environmental justice index (EJI). Agency for toxic substances and disease registry (ATSDR) (2024). Available online at: https://www.atsdr.cdc.gov/place-health/php/eji/index.html (Accessed May 30, 2025). 10.3109/15360288.2015.1037530 26095483

[4] Yilma M Cogan R Shui AM Neuhaus JM Light C Braun H Community-level social vulnerability and individual socioeconomic status on liver transplant referral outcome. Hepatol Commun. (2023) 7(7):e00196. 10.1097/HC9.0000000000000196 37378636 PMC10309511

[5] Hasjim BJ Huang AA Paukner M Polineni P Harris A Mohammadi M Where you live matters: area deprivation predicts poor survival and liver transplant waitlisting. Am J Transplant. (2024) 24(5):803–17. 10.1016/j.ajt.2024.02.009 38346498 PMC11070293

[6] Buggs J Patino D Evans B Anderson C Delfavero C Rogers E The role of non-traditional environmental factors in long-term outcomes of liver transplantation. Am J Transplant. (2019) 19(Suppl 3). https://atcmeetingabstracts.com/abstract/the-role-of-non-traditional-environmental-factors-in-long-term-outcomes-of-liver-transplantation/

[7] Adhikari A Nwosu A Qian M Hellegers C Devanand DP Doraiswamy PM. Characterizing neighborhood vulnerabilities in mild cognitive impairment using the environmental justice index. J Alzheimers Dis Rep. (2024) 8(1):793–804. 10.3233/ADR-240020 38910939 PMC11191642

[8] Snider-Hoy NG Buchalter RB Hastert TA Dyson G Gronlund C Ruterbusch JJ Social-Environmental burden is associated with increased colorectal cancer mortality in metropolitan detroit. Cancer Res Commun. (2025) 5(4):694–705. 10.1158/2767-9764.CRC-24-0503 40293949 PMC12036821

[9] Fairless BM Fawole O Nguyen DT Banerjee A Nobleza KJ Oluyomi AO Neighborhood disadvantage and inequities in access to preemptive and living kidney transplantation. Kidney360. (2025) 6(6):1007–19. 10.34067/KID.0000000724 40117583 PMC12233847

[10] Chakraborty J Collins TW Grineski SE Montgomery MC Hernandez M. Comparing disproportionate exposure to acute and chronic pollution risks: a case study in Houston, Texas. Risk Anal. (2014) 34(11):2005–20. 10.1111/risa.12224 24913274

[11] Whitworth KW Moussa I Salihu HM Chardon Fabien A Suter M Aagaard KM Environmental justice burden and black-white disparities in spontaneous preterm birth in Harris county, Texas. Front Reprod Health. (2023) 5:1296590. 10.3389/frph.2023.1296590 38179111 PMC10766384

[12] American Public Health Association (APHA). Environmental justice index (EJI): understanding the role of environmental burden in health. Available online at: https://www.apha.org/getcontentasset/78f74b83-ed4d-4835-b032-2590dbe7b118/7ca0dc9d-611d-46e2-9fd3-26a4c03ddcbb/ejiwebinar_presentationslides.pdf (Accessed May 30, 2025).

[13] U.S. Department of Health and Human Services. Environmental justice index (EJI): Frequently asked questions. agency for toxic substances and disease registry (ATSDR). Available online at: https://www.atsdr.cdc.gov/place-health/php/eji/eji-frequently-asked-questions.html (Accessed May 30, 2025).

[14] Agency for Toxic Substances and Disease Registry (ATSDR). *Environmental Justice Index (EJI) Community Engagement Report*. U.S. Department of Health and Human Services (2024). Available online at: https://www.atsdr.cdc.gov/place-health/media/pdfs/2024/10/EJI-Community-Engagement-Report-508.pdf (Accessed June 2, 2025).

[15] U.S. Department of Health and Human Services. Social vulnerability Index (SVI). Agency for Toxic Substances and Disease Registry (ATSDR) (2024). Available online at: https://www.atsdr.cdc.gov/place-health/php/svi/index.html (Accessed May 30, 2025). 10.3109/15360288.2015.1037530 26095483

[16] U.S. Department of Health and Human Services. Social determinants of health among transplant patients data report now available. Organ Procurement and Transplantation Network (OPTN). (2024). Available online at: https://optn.transplant.hrsa.gov/news/social-determinants-of-health-among-transplant-patients-data-report-now-available (Accessed May 30, 2025). 10.3109/15360288.2015.1037530 26095483

[17] Osakwe NC Motsinger-Reif AA Reif DM. Environmental health and justice screening tools: a critical examination and path forward. Front Environ Health. (2024) 3:1427495. 10.3389/fenvh.2024.1427495

[18] Suarez-Pierre A Iguidbashian J Kirsch MJ Cotton JL Quinn C Fullerton DA Importance of social vulnerability on long-term outcomes after heart transplantation. Am J Transplant. (2023) 23(10):1580–9. 10.1016/j.ajt.2023.06.017 37414250

[19] Park C Schappe T Peskoe S Mohottige D Chan NW Bhavsar NA A comparison of deprivation indices and application to transplant populations. Am J Transplant. (2023) 23(3):377–86. 10.1016/j.ajt.2022.11.018 36695687 PMC10226151

[20] Grunwell JR Mutic AD Ezhuthachan ID Mason C Tidwell M Caldwell C Environmental injustice is associated with poorer asthma outcomes in school-age children with asthma in metropolitan Atlanta, Georgia. J Allergy Clin Immunol Pract. (2024) 12(5):1263–72.e1. 10.1016/j.jaip.2024.02.015 38378096 PMC11081836

[21] Ashad-Bishop KC Wiese D Baeker Bispo JA Ogongo MK Islami F Bandi P. Social-Environmental injustice and cancer screening prevalence. JAMA Netw Open. (2024) 7(9):e2433724. 10.1001/jamanetworkopen.2024.33724 39283641 PMC11406390

[22] Kodali S Mobley CM Brombosz EW Lopez A Graves R Ontiveros J Effect of a hispanic outreach program on referral and liver transplantation volume at a single center. Transpl Immunol. (2024) 84:102034. 10.1016/j.trim.2024.102034 38499048

[23] Rea KE West KB Dorste A Christofferson ES Lefkowitz D Mudd E A systematic review of social determinants of health in pediatric organ transplant outcomes. Pediatr Transplant. (2023) 27(1):e14418. 10.1111/petr.14418 36321186

[24] Henson JB Chan NW Wilder JM Muir AJ McElroy LM. Characterization of social determinants of health of a liver transplant referral population. Liver Transpl. (2023) 29(11):1161–71. 10.1097/LVT.0000000000000127 36929783 PMC10509317

