# Supplementary figures and images for: Evaluating the impact of neighborhood socioenvironmental burden on patient characteristics and survival in liver transplant recipients
Source: Front Transplant. 2026 Apr 9;5:1731241. doi: 10.3389/frtra.2026.1731241 (PMC13102850; doi:10.3389/frtra.2026.1731241)

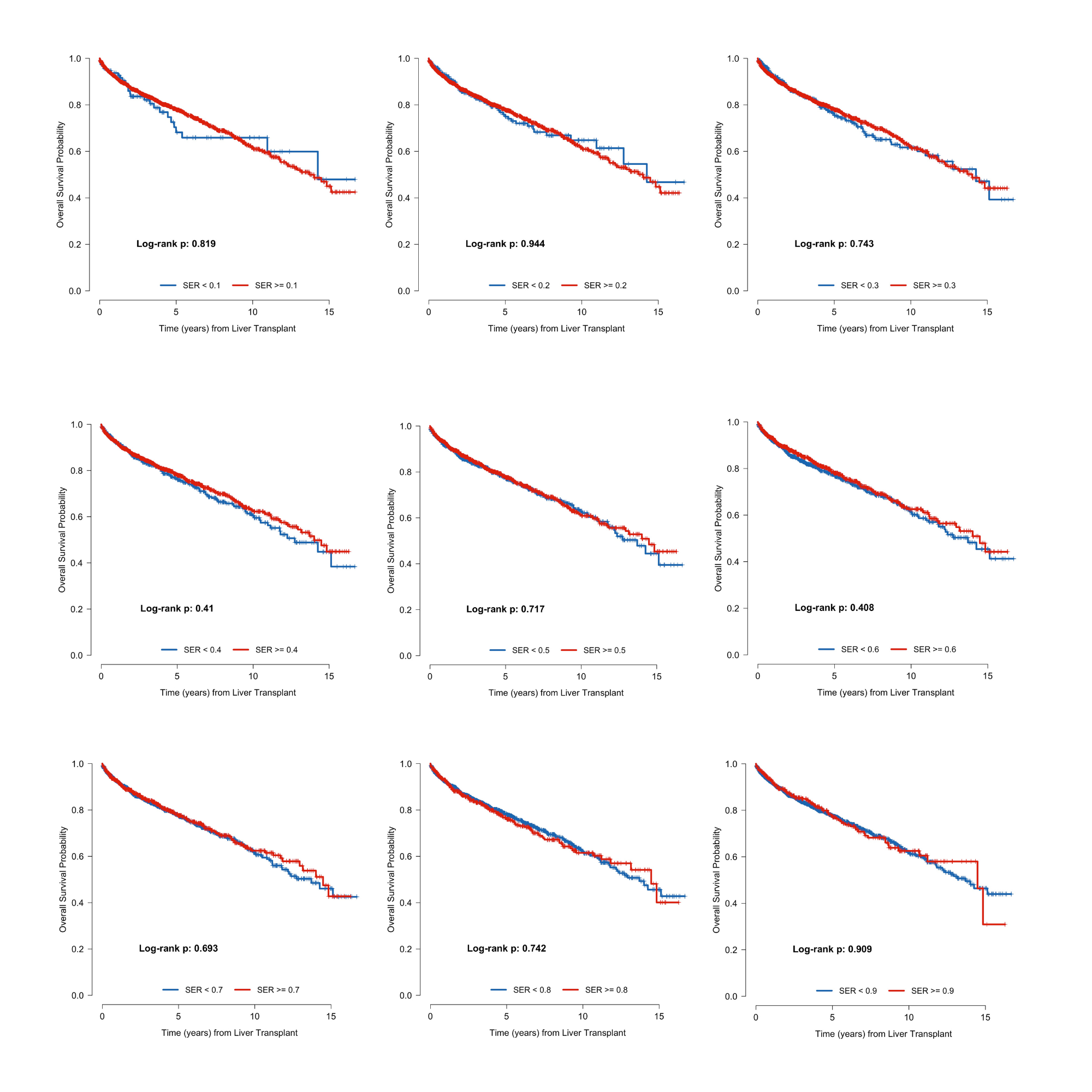

Supplement: Supplementary file 1 [file Image1.png]
